# Market Failure and the Poverty of New Drugs in Maternal Health

**DOI:** 10.1371/journal.pmed.0050022

**Published:** 2008-01-22

**Authors:** Nicholas M Fisk, Rifat Atun

## Abstract

A new survey finds that only 17 drugs are under active development for maternal health indications, which is less than 3% of the pipeline in cardiovascular health (660 drugs).

The pharmaceutical industry's business model is hefty investment in research and development (R&D), in expectation of high returns from future drug sales during the period of patent protection This model, which funds around 50% of health care R&D in the United States and a higher proportion in Europe [1], generates 20–25 new licensed drugs per year, but very few for use in pregnancy.

After thalidomide and diethylstilboestrol, risk of teratogenicity has led to understandable caution in developing drugs for pregnancy and including women in clinical trials, but this has meant increased off-label use, with 75% of pregnant women taking at least one drug for which safety data are unavailable [2]. A greater problem is the dearth of drugs developed specifically for obstetric conditions. With the exception of abortifacients and reformulations, only three new drugs (atosiban, carboprost, and carbetocin) have been licensed over the last two decades in the United Kingdom for obstetric indications, two of which are only used after delivery. In the US, no licensed drug is available for use in preterm labour. No new classes of drug have been developed for the big diseases of preeclampsia, fetal growth restriction, postpartum haemorrhage, and miscarriage [3,4]. The mainstays of the 2007 obstetric formulary (magnesium sulfate, α-methyldopa, hydralazine, β-blockers, aspirin, and nifedipine) hark back to an earlier era, and give resonance to Archie Cochrane's 1979 award of the “wooden spoon” to obstetrics as the least scientific specialty in medicine [5].

The paucity of obstetric drugs impacts not only the resource-rich countries, but also affects the far greater disease burden in resource-poor settings. Maternal and perinatal conditions are the single largest contributor to the global burden of disease, accounting for 6% of disability-adjusted life years (DALYs) [6], and would account for more if stillbirths were not excluded. Worldwide, there are 536,000 maternal deaths annually [7], while nearly half the 13.5 million under-five child deaths occur as antepartum, intrapartum, or neonatal deaths [6,8]. These are disproportionately concentrated in the developing world, where 99% of maternal deaths occur, three-quarters due to preventable or treatable conditions such as haemorrhage, hypertensive disorders of pregnancy, sepsis, obstructed labour, and unsafe abortion [9]. Two of the United Nations General Assembly's eight Millennium Development Goals (MDGs 4 and 5) set targets for reducing maternal and under-five child mortality rates by 3/4 and 2/3 respectively between 1990–2015 [10]. However, progress in reducing maternal and perinatal mortality has been disappointing, and these targets are unlikely to be met [11].

**Summary Box**

- The existing R&D and business model of the pharmaceutical industry has failed to develop drugs for obstetric conditions, with only one new class of drug licensed in the past 20 years, and no new class of drug in clinical trials for primary obstetric applications.
- Only 17 drugs are under active development for maternal health indications; less than 3% of the pipeline in cardiovascular health (660 drugs) and fewer than for a single rare disease like amyotrophic lateral sclerosis (34 drugs).
- Reasons for the paucity of new drugs for obstetrics include risk aversion, the cost of reproductive toxicology studies, the small market size, and a weak regulatory system, which encourages endemic off-label use.
- Obstetric pathologies have great impact on maternal and perinatal disease burden in the developing world, yet the paucity of drugs for obstetric disease has attracted little attention from international donor agencies.
- We document the global drug drought in maternal health to conclude that the market has failed pregnant women.

The eighth MDG encourages global partnerships to “in co-operation with pharmaceutical companies, provide access to affordable essential drugs in developing countries” [10], including those for neglected diseases for which there is not a large enough market to attract private sector investment in R&D. A number of instruments to achieve this have been developed by national governments and internationally (Box 1). Whereas *push* mechanisms provide funding to encourage universities, small-medium enterprises, and multinational pharmaceutical companies to invest in R&D to generate new drugs for neglected diseases in developing countries (commercially unattractive due to small market size), *pull* mechanisms provide funds to purchase these drugs or vaccines once they come to market, or signal in advance a price or the presence of a “market” for those chemical entities for neglected diseases that are in R&D pipelines. Public–private product development partnerships (PPPDPs) have been the preferred route for push mechanisms and by 2005 had attracted US$212 million of philanthropic and US$44 million of public funding. Early evidence suggests increased R&D activity in infectious and tropical diseases: while only 13 new drugs for neglected diseases were developed between 1975–1997 [12], by 2004 there were 63 R&D projects supported by PPPDPs with 20 drugs in clinical trials or registration, and eight to nine new drugs anticipated to market by 2010 [13].

However, in spite of record investment in pharmaceutical R&D in rich countries, and global initiatives to address disease burden in developing countries, there is still a paucity of new chemical entities for diseases of pregnancy. So have the market models for pharmaceuticals in developed countries and international push and pull mechanisms to create artificial markets for otherwise unaffordable drugs and vaccines in developing countries failed for maternal health? This study investigates the current obstetric R&D pipeline.

## Methods

We undertook a search of the Pharmaprojects database (http://www.pharmaprojects.com/). This industry database used by over 700 pharmaceutical companies tracks all drugs identified as being under development from Web sites, conferences, PubMed, and registered clinical trials (over 37,000 listed since 1981). Although early pre-clinical capture is incomplete, late pre-clinical and clinical coverage is comprehensive, with around 10% of listed drugs eventually being licensed.

To identify the status of each drug in the R&D pipeline over the period from 1980–2007, we searched for the terms pre-eclampsia, eclampsia, pregnancy, pregnancy-induced hypertension, preterm/premature labour/labour, tocolysis or tocolytic, threatened or recurrent miscarriage, preterm ruptured membranes, intrauterine or fetal growth restriction, induction, inhibition or augmentation of labour, oxytocic, postpartum haemorrhage, post-maturity, anaemia in pregnancy, obstetric cholestasis, alloimmunisation, or hyperemesis gravidarum. Individual project files were then reviewed to identify drugs with primary or secondary obstetric applications, their class and regulatory status, and whether they were new chemical entities (also known as new molecular entities in the US, or new active substances in the European Union) or reformulations.

We also searched the Pharmaprojects database for comparator sectors/diseases to illustrate relative activity—we chose to examine drugs for cardiovascular indications and for amyotrophic lateral sclerosis, a rare condition affecting two to five of 100,000 people [14]. The first was chosen as a mainstream but not leading specialty area for pharmaceutical R&D, having less than half the drugs in development as cancer, and less than 60% of those in neurology [15]. Amyotrophic lateral sclerosis was selected because of its low market potential to generate revenues (only 20,000 patients in the seven main markets), such that it attracts orphan disease status in both Europe and the US.

Our database search was augmented by (a) PubMed searches to identify any previous articles on this topic, articles on licensed and/or unlicensed drug use in obstetrics, and articles on licensed and/or unlicensed drug use in child health for comparison; (b) specific searches for the above on the official Web sites of the US Food and Drug Administration (FDA; http://www.fda.gov/), the European Medicines Agency (http://www.emea.europa.eu/), the UK Medicines and Healthcare Products Regulatory Agency (http://www.mhra.gov.uk/), the World Health Organization (WHO; http://www.who.int/), the International Federation of Pharmaceutical Manufacturers & Associations (IFPMA; http://www.ifpma.org/), the Pharmaceutical Research and Manufacturers of America (PhRMA; http://www.phrma.org/), and the Association of the British Pharmaceutical Industry (ABPI; http://www.abpi.org.uk/), as well as key international PPPDPs (Box 1); (c) search of drugs listed under obstetric indications in the British National Formulary (http://www.bnf.org/) against registration dates listed in the Electronic Medicines Compendium (http://emc.medicines.org.uk/); (d) search of the IFPMA's Clinical Trials Portal (http://clinicaltrials.ifpma.org/), the PhRMA's Clinical Study Results Database (http://www.clinicalstudyresults.org/), and the US National Institutes of Health's Clinical Trials Database (http://clinicaltrials.gov/), using the same search terms as for Pharmaprojects; and (e) discussions with opinion leaders/senior clinical investigators in each of the main therapeutic areas in obstetrics (preterm labour, hypertensive disease of pregnancy, fetal growth restriction, intrapartum care, miscarriage).

## Results

Overall, 67 drugs were listed in Pharmaprojects for maternal indications, mainly for preterm labour (45%, 30 out of 67). There were 17 drugs under active development as of November 2007. Only three were in pre-clinical studies, and 13 were in clinical trials (five in phase I, five in phase II, and three in phase III), with a further one awaiting registration. Nine of 10 drugs listed as licensed had been launched since 1981, but only three were licensed in both Europe and the US; two were formulations of dinoprostone, a prostaglandin E2 for labour induction, and the third was a prostaglandin F2a analogue for post-partum haemorrhage. Four were licensed in Japan and/or Korea only (all for labour induction: two sodium prasterone formulations, one prostaglandin E2 reformulation, and one oxytocin agonist). The only new class of drug listed (atosiban, an oxytocin receptor antagonist) was licensed in Europe but not the US. In 40 cases, drugs were listed as discontinued, suspended, or inactive.

Table 1 shows the indications and status of drugs under development in maternal health. Only nine of the 17 were new chemical entities, eight for preterm labour, with one an antithrombotic against the antiphospholipid syndrome with potential application in early pregnancy. Four of the six new chemical entities developed specifically for obstetrics are in clinical trials, all second-generation oxytocin antagonists. Thus there are only three new chemical entities in pre-clinical and no new class of drug in clinical trials for primary indications in pregnancy. The single drug in pre-registration and all three in phase III (all misoprostol or progesterone) arose from academic research rather than pharmaceutical R&D, pharmaceutical entry occurring late in development with generic reformulation.

**Table 1:**
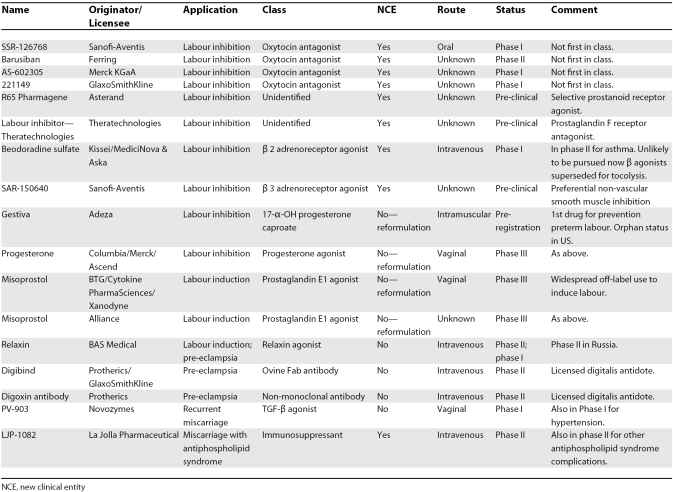
Details of Drugs Under Active Development for Maternal Health Indications

Because it takes 10–15 years on average across all therapeutic areas to develop and launch a new drug [15], it is possible to compare the number of drugs in development between therapeutic categories. The 17 drugs currently under development in obstetrics compare with 660 drugs in a mainstream therapeutic area like cardiovascular disease (Table 2). There were more drugs currently under development for amyotrophic lateral sclerosis than for all obstetric applications (34 versus 17) (Table 2). Overall, 310 compounds were listed as orphan drugs under development, only one of which was obstetric.

**Table 2:**
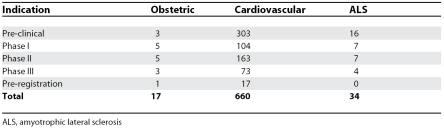
Comparison of the Obstetric Drug Pipeline with that of a Mainstream Area (Cardiovascular) and that of a Neglected Disease (Amyotrophic Lateral Sclerosis)

To confirm complete ascertainment of the obstetric drug pipeline, we reviewed recent publications and clinical trials databases maintained by the pharmaceutical industry. Industry publications in 2006–2007 listing medicines in R&D, such as the ABPI's A–Z of Medicines Research and the PhRMA's Industry Profile, make no mention of any obstetric drugs [1,15]. Our search revealed no obstetric trials within the PhRMA's Clinical Study Results Database (http://www.clinicalstudyresults.org/). Search of the broader IFPMA Clinical Trials Portal (http://clinicaltrials.ifpma.org/) and the National Institutes of Health's Clinical Trials Database (http://clinicaltrials.gov/) found only seven of the industry-sponsored drugs displayed in Table 1; five other drugs were listed. In three (all reformulations of older drugs), the pharmaceutical partner was not listed as the primary sponsor; a further one (sildenafil for pre-eclampsia) was listed in the Pharmaprojects database as having development recently suspended, while one plant extract was in phase II/III sponsored by a complementary medicines developer. Numerous investigator-led trials (more than 50) were listed, usually of agents licensed for other indications and/or already used in obstetrics, but none primarily sponsored by the pharmaceutical industry were identified that are not shown in Table 1. Our belts-and-braces strategy of searching an industry drug development database complemented by Web searches of national and regional registration bodies, industry associations, and clinical trials databases was designed to maximize ascertainment, but we acknowledge that it might have missed some agents. Nonetheless, we were unable to identify any obstetric drugs being developed by the pharmaceutical industry additional to those listed in Table 1.

**Box 1. Key Push and Pull Mechanisms Relevant to MDG 8**

**Push Mechanisms**

- A range of instruments used by the US FDA to promote drug development, such as patent extensions to encourage licensing of drugs in children, tax incentives to promote development of drugs granted orphan status (for drugs with US market size <200,000 patients), and fast-track options for approving drugs in areas of unmet medical need (http://www.fda.gov/).
- The UK Department of Health's Medicines for Children Initiative, which provides financial support to research networks for developing paediatric drugs [34].
- The WHO Special Programme for Research and Training in Tropical Diseases (WHO-TDR), established in 1970, is a partnership of four cosponsors, 22 WHO member states, and 12 foundations and agencies with annual funds of US$30 million (http://www.who.int/tdr/index.html).
- The Medicines for Malaria Venture, founded in 1999, has attracted US$263 million from donors (http://www.mmv.org/).
- The TB Alliance, founded in 2000, has attracted US$193 million in funding (http://www.tballiance.org/).
- The Institute for OneWorld Health founded in 2000, a US-based non-profit pharmaceutical company (http://www.oneworldhealth.org/).
- The Drugs for Neglected Diseases Initiative founded in 2003 by five public-sector research organisations, Médecins Sans Frontières, and WHO-TDR (http://www.dndi.org/).
- The GAVI Alliance, a global PPPDP, which supports R&D and access to vaccines for children in developing countries (http://www.gavialliance.org/).
- The International AIDS Vaccine Initiative (http://www.iavi.org/), a global PPPDP, which supports R&D to develop AIDS vaccines.

**Pull Mechanisms**

- 2007 EU Regulations on Paediatric Medicines, which provide for market exclusivity extensions for drugs designed for paediatrics (http://www.mhra.gov.uk/).
- Supplementary protection certificates in Europe provide patent extensions (once the corresponding patent expires) of up to five years for drugs with a new ingredient, to compensate for regulatory delays during development and approval stages [35].
- Advanced market commitments, aimed at creating a market for future vaccines to stimulate private investment in vaccine R&D and manufacturing capacity, are legally binding commitments to purchase at an agreed price vaccines once developed that primarily address diseases of resource-poor countries [32]. This is financed through the International Finance Facility for Immunisation, a multilateral organisation supported by several sovereign donors to the tune of US$4 billion.
- The Global Fund to Fight AIDS, Tuberculosis and Malaria, which since 2001 has committed US$7.1 billion in 136 countries (in part for drug purchase to control these diseases) (http://www.theglobalfund.org/).
- PEPFAR (US President's Emergency Plan for AIDS Relief), which by September 2006 had provided US$819 million for purchase of antiretroviral drugs for HIV (http://www.pepfar.gov/).
- UNITAID uses airline taxes to create a market for missing essential medicines, such as paediatric medicines for HIV/AIDS and second-line medicines for tuberculosis and malaria (http://www.unitaid.eu/en/).
- Social policy bonds, defined as “non-interest bearing bonds, redeemable for a fixed sum only when a targeted social objective has been achieved”, could be an alternative to advance market mechanisms, and could be used to signal substantial incentives for success in achieving a social policy goal of developing an effective obstetric drug and using it to reduce maternal or perinatal mortality in a particular setting [33].

## Discussion

We demonstrate significant under-investment by pharmaceutical companies in maternal health and the existence of a drug drought in obstetric therapeutics. Only one new class of drug has been licensed for obstetric conditions in the last 20 years, and the situation is set to worsen, with no new class of drug developed primarily for obstetric applications in clinical trials, and only three new chemical entities in pre-clinical. The meagre obstetric drug pipeline represents less than 3% of the activity (by number of drugs under investigation) in cardiovascular health, and considerably less than the activity for a single orphan disease. Pregnancy has become a virtual “pharma-free zone”, with a recent 137-page industry review of medicines development failing to mention any potential application in pregnancy [1]. Our findings systematically demonstrate a “pharmaceutical gap” in medicines for women, whose needs, according to the WHO report on Priority Medicines for Europe, have often been neglected by manufacturers and regulators [16].

Pregnant women look set to miss out on the therapeutic advances expected from modern drug R&D in other fields that will benefit from combinatorial chemistry, high throughput screening, pharmacogenomics, bioinformatics, nanotechnology, the “-omic” sciences, and biologics. The market economy approach has largely abandoned progress in obstetric therapeutics to the feebler efforts of investigator-driven research and unlicensed usage. Most of the everyday drugs we now take for granted in other fields, e.g., ulcers, arthritis, heart conditions, etc., would not exist if they too had been left to the exigencies of R&D from the public purse. Curiosity-driven research in maternal and perinatal health has made limited progress in understanding the aetiology and pathophysiology of most complications of pregnancy. Similarly, investigator-driven drug trials of existing agents can have an impact on therapeutic practice (e.g., magnesium sulfate for pre-eclampsia [17]), but are most unlikely to develop much needed novel therapeutic agents. Consequently, clinical management has focused on physical treatments such as caesarean section, induction of labour, and neonatal intensive care rather than pharmaceutical preventive and treatment strategies.

While improving basic living conditions with access to good obstetric services and existing drugs will undoubtedly help reduce a large proportion of maternal and perinatal deaths in developing countries, there remains a substantial burden of disease unaddressed by the existing portfolio of drugs. In 2007, the treatment for two of the three chief obstetric pathologies remains expediting delivery (pre-eclampsia and growth restriction), while the third (preterm labour) is managed by crude attempts at stopping labour with drugs of questionable efficiency. Yet each pathology carries a substantial disease burden affecting mother and baby. Indeed, these conditions are the three main contributors to low birth weight, responsible for over 3% of global DALYs [16].

New drugs could improve effective management of pre-eclampsia, growth restriction, and pre-term labour globally, while wider use of existing drugs (e.g., magnesium sulfate for pre-eclampsia, which remains unavailable in many developing countries) [18], and improved formulation of available drugs could substantially reduce maternal deaths in developing countries. For example, intravenous oxytocin, which effectively treats postpartum haemorrhage (the cause of 25% of maternal deaths), requires refrigeration for storage, which prevents its use in developing countries that lack such resources. Despite WHO studies in 1993 and 1994 showing loss of potency of oxytocic drugs in field conditions, no heat-stable and simple-to-use oxytocin injection has been developed [16].

Although this report focuses on drugs for primary obstetric indications, it raises additional concerns about widespread off-label use of medicines in women. The high cost of additional clinical trials and litigation risks discourage testing (for safety and efficacy) in pregnant women of drugs proven to be safe in non-pregnant adult populations. While the lack of data on medication in pregnancy has been a safety concern for many decades, this area remains under-funded [16].

### Reasons for market failure.

There are a number of reasons for this market failure, all of them addressable. The first is the reluctance of pharmaceutical companies to test (let alone develop) drugs in pregnancy, in part because of the additional cost of reproductive toxicology studies, but mainly because of risk aversion to the possibility of teratogenicity. The latter is exacerbated by high lifelong settlement costs for a baby damaged in utero (up to £5m in the UK), and in the US by a jury-determined tort process, which favours punitive damages (up to US$110 million). Safety in human pregnancy can really only be assured by post-marketing surveillance, it taking a mean of six years to identify teratogenicity in 11 such drugs approved between 1980 and 2000 [19], which creates additional and increasingly prohibitive cost. However, although such commercial barriers to entry are understandable, teratogenicity is really only an issue in early pregnancy and of little relevance to drug development in later pregnancy, when there is a genuine unmet need in treating pre-eclampsia, preterm labour, growth restriction, and the complications of labour. Non-teratological serious adverse outcomes may still prove a regulatory and litigation risk, as neurological sequelae in the preterm infant can require decades of support.

The second reason is the small market size for conditions affecting pregnant women. Highest returns in the biopharmaceutical sector typically come from drugs taken life-long for chronic diseases with good life expectancy. In contrast, pregnancy is short-term, the number of children per women is in decline, and most obstetric pathologies are short-lived. Global markets are thus estimated in the lower end of the US$0–US$500 million range, although this could rise many-fold with development of prophylaxis against preterm labour or pre-eclampsia. Nevertheless, there are still around 4.1 million births annually in the US and 4.8 million in the EU [20,21]. Around 2% of pregnant women will deliver =32 weeks after preterm labour, 3%–5% will develop pre-eclampsia, 2%–3% will develop problematic growth restriction, 20% will require induction of labour, and 0.5% will develop massive postpartum haemorrhage. Drugs have been successfully developed for acute conditions in other fields and for much smaller market sizes.

The third reason is the industry model. The limitations of the shareholder model for drug development for neglected and rare diseases are acknowledged, as are the efforts made to address market failure for these diseases. However, the disease frequencies outlined above and the untapped developed world market suggests reasonable potential returns in obstetrics. The high degree of regulation of drug safety in pregnancy together with the paucity of pipeline drugs creates greater potential for revenue shocks, to which the industry is averse. High-profile product withdrawals leading to rapid falls in share price and revenues, increasing litigation, stronger regulation, the rising cost and complexity of R&D from new technologies, and high costs of commercialisation and post-marketing surveillance have encouraged risk aversion, with pursuit of blockbuster drugs (annual sales exceeding US$1 billion) at the expense of solutions for disease groups that have smaller market potential [22]. This contributed to the fall in new drug approvals in 2006 to 18, the second lowest number ever [23]. These factors deter investment in a risky, highly regulated, small market segment like obstetrics.

The fourth reason is gaps in the regulatory system. Most drugs used antenatally are unlicensed in pregnancy. An example is the use of betamethasone to promote fetal lung maturity, the only drug proven to reduce perinatal mortality [24]. A system that allows off-label use has advantages in accessing drugs for rare diseases and situations with an emerging evidence base, but becomes a major disadvantage where long-term off-label use becomes endemic, as it discourages pharmaceutical investment in clinical trials. This is clearly unacceptable, as lack of evidence on safety or efficacy places women at risk. The resultant impoverished evidence base creates additional regulatory risks for new entrants into the obstetric market. These risks are illustrated by the experience of the only company developing a portfolio in obstetrics, in licensing its oxytocin receptor antagonist [25]. FDA approval was not pursued because of the lack of effect of tocolytics overall on perinatal morbidity and mortality in placebo-controlled trials [26], while in Europe post-approval sales were eroded by off-label competition from a generic calcium antagonist. Widespread use of unlicensed drugs, or rather the off-label obstetric use of drugs licensed for non-obstetric indications, is analogous to the situation in paediatrics and neonatology [27], where as a consequence there is uncertainty about the safety, dosage, and efficacy of drugs in everyday use.

### Push, pull, and fail.

Health gains from new medicines eventually benefit the developing world (although often not until they come off patent), but in obstetrics the paucity of new drugs minimises this trickle-down effect. In terms of global disease burden, besides improving access to obstetric services, investing in drugs for postpartum haemorrhage and pre-eclampsia, key contributors to avoidable pregnancy mortality [28], would contribute towards the reduction of death rates in pregnancy and our chances of achieving a key MDG target.

Attempts to alleviate the drug drought in maternal health will need both to create incentives for investment in pharmaceutical R&D and to attract the attention of the international donor agencies. The various push and pull mechanisms to date have focused on vaccines and neglected diseases, with little evidence of any interest in maternal health. Regulatory gaps must be plugged to reduce off-label use. In parallel, new mechanisms and incentives must be put in place to encourage systematic testing (for safety and efficacy) of existing and new drugs for indications that are relevant to pregnancy and obstetric practice, especially those for life-threatening conditions (such as severe infections) that also affect pregnant women.

While there is some evidence of progress with paediatric medicines (another area that has traditionally attracted low R&D investment) in both the US and the UK with specific initiatives and regulatory concessions [16,29,30,31], there are none for women. Such regulatory concessions could be considered to reduce commercial risks inherent in developing obstetric drugs.

Models successfully used as “push” mechanisms to encourage investment in, and development of drugs for, neglected diseases with promising pipelines, such as the Medicines for Malaria Venture (http://www.mmv.org/), the TB Alliance (http://www.tballiance.org/), the Drugs for Neglected Diseases Initiative (http://www.dndi.org/), and the GAVI Alliance (http://www.gavialliance.org/), could be replicated to provide dedicated funds for R&D in maternal health. Alternatively, models such as advanced market commitments [32] or the Global Fund to Fight AIDS, Tuberculosis and Malaria (http://www.theglobalfund.org/) could be used to create a global “pull” mechanism and to signal a viable market for these drugs to encourage investment in obstetric drug R&D. A further option would be to create a not-for-profit entity, such as the Institute for OneWorld Health (http://www.oneworldhealth.org/), dedicated specifically to maternal health (Box 1). These are well-developed models with some indication of success, but none have been forthcoming for maternal health. In particular, the not-for-profit option, which unlike other models does not rely on profit for innovation, should be carefully assessed. Further innovative approaches, for example social policy bonds (issued by governments, the private sector, and other organisations as promissory notes to be paid upon achieving a target) could be used to create further incentives to complement push and pull mechanisms or reward not-for-profit organisations [33].

The market has failed pregnant women. Between the *pull* and the *push*, the international donor agencies have also forgotten these women. Given the unacceptably high number of maternal and perinatal deaths each year, it is high time to address this failure.

## Abbreviations

ABPI: Association of the British Pharmaceutical Industry
DALY: disability-adjusted life year
FDA: Food and Drug Administration
MDG: Millennium Development Goal
IFPMA: International Federation of Pharmaceutical Manufacturers & Associations
PhRMA: Pharmaceutical Research and Manufacturers of America
PPPDP: public–private product development partnerships
R&D: research and development
WHO: World Health Organization
